# Gene variant C3 glomerulonephritis with chronic urinary tract infection: A case report and literature review

**DOI:** 10.1097/MD.0000000000041001

**Published:** 2024-12-27

**Authors:** Chao Xu, Junfen Fan

**Affiliations:** a Department of Nephrology, The First Affiliated Hospital of Zhejiang Chinese Medical University (Zhejiang Provincial Hospital of Chinese Medicine), Hangzhou, Zhejiang, PR China.

**Keywords:** C3 glomerulonephritis, C3 glomerulopathy, case report, CD46, chronic urinary tract infection, complement C3

## Abstract

**Rationale:**

C3 glomerulonephritis (C3GN) is 1 type of C3 nephropathy which is a rare glomerular disease associated with abnormal regulation of the alternative complement pathway. This review reports a rare case of C3GN with repeated urinary tract infection and summarizes the clinical features, differential diagnosis, treatment, and outcome of patients with C3GN.

**Patient concerns:**

A 44-year-old woman was admitted to our hospital because of proteinuria increased for more than 8 years.

**Diagnoses:**

The patient was finally diagnosed with C3GN by renal biopsy and gene testing.

**Interventions:**

The patient was worried about the side effects of drugs and strongly refused to use glucocorticoids and immunosuppressants, so she was given angiotensin II receptor blocker monotherapy for glomerulonephritis and quinolones for urinary tract infection.

**Outcomes:**

Serum creatinine, proteinuria, and serum complement c3 levels remained stable. Our case is still under continuous follow-up.

**Lessons:**

Clinical diagnosis of C3GN is difficult to make as there are many differential diagnoses, especially post infection nephritis. This case emphasizes the importance of renal biopsy in the diagnosis of C3GN, but the relationship between gram-negative bacilli and C3GN is still unclear. In addition, gene mutation is also involved in the pathogenesis of C3GN, and the treatment of C3GN still needs to be explored.

## 1. Introduction

C3 nephropathy (C3G) is a kind of disease which has been reclassified in recent years. It is defined as a kind of kidney disease with only C3 deposition or weak positive deposition of immunoglobulin and/or Clq in renal tissue, including C3 glomerulonephritis (C3GN) and dense deposition disease (DDD).^[1]^ All C3G except DDD are classified as C3GN, including type I membranoproliferative glomerulonephritis (MPGN) and complement H factor related protein 5 nephropathy which are mainly C3 deposition, familial type III MPGN, etc. C3GN was first reported by VB in 1974.^[2]^ It was described that only complement C3 was deposited, and immunoglobulin and C1q were negative. Due to the lack of deposition of classical complement pathway and lectin pathway related components (such as immunoglobulin, C4, Clq, etc), it is suggested that C3G is related to the abnormal activation of complement pathway.

It is well known that infection can activate complement system, and then lead to C3GN. In the diagnosis of C3GN, it needs to be differentiated from postinfectious glomerulonephritis. In the reported literature, we found that most postinfectious nephritis is caused by gram-positive bacteria. There is no report of gram-negative bacteria causing C3GN. We find that the gram-negative bacteria associated urinary tract infection are related to C3GN in this case.

## 2. Case presentation

A 44-years-old woman who is a music teacher was admitted to the First Affiliated Hospital of Zhejiang University of Traditional Chinese Medicine with Proteinuria increased for more than 8 years. Eight years ago the patient found that urine foam increased during pregnancy. Urinalysis revealed proteinuria (2+), serum creatinine value was in normal range, no hypertension, no hematuria, after non glucocorticoid therapy proteinuria turned to negative. The pregnancy was terminated automatically. Six years ago, in order to have a tube baby the patient took a glucocorticoid therapy, conducted obvious side effects including edema, ascites, and relieved after withdrawal. During that time, it was found that serum C3 was low, proteinuria continued, and no treatment was taken. In 2017, the patient became pregnant again, and the proteinuria was obviously increased during pregnancy. Urinalysis revealed proteinuria (3+), serum creatinine value was still in normal range. After receiving non-glucocorticoid therapy, the urine protein maintained (1+) and gave birth to another daughter. The patient has not gone to the hospital since then. The patient’s serum creatinine maintained normal range so far and she complained of repeated urinary tract infection. She took quinolones when felt uncomfortable in urinating, and stopped immediately when symptoms relieved. There was a history of upper respiratory tract infection 2 weeks before hospitalization and relieved after oral antibiotics. After hospitalization, the patient still had sore throat, cough, no phlegm, and no fever. Testing shows urine protein (+)–(2+), occult blood (3+), nitrite (2+), urine culture showed *Escherichia coli* resistance to quinolones, complement C3 0.15 g/L (normal range: 0.79–1.52 g/L). The 24-hour urine protein was 1500 mg (normal range: 0.0–150 mg) and the urine volume was 2500 mL. The proportion of abnormal red blood cells was 75%. Microalbumin was 1048.8 mg/L (normal range: 0.00–30.0 g/L), microalbuminuria creatinine ratio was 0.973 mg/mgcr (normal range: 0.000–0.030 mg/mgcr). Ophthalmic examination shows nothing abnormal. Hepatitis, HIV and syphilis were negative, IgA, IgG, IgM, C4 were in normal range and the blood light chain was in normal range.

**Figure 1.**
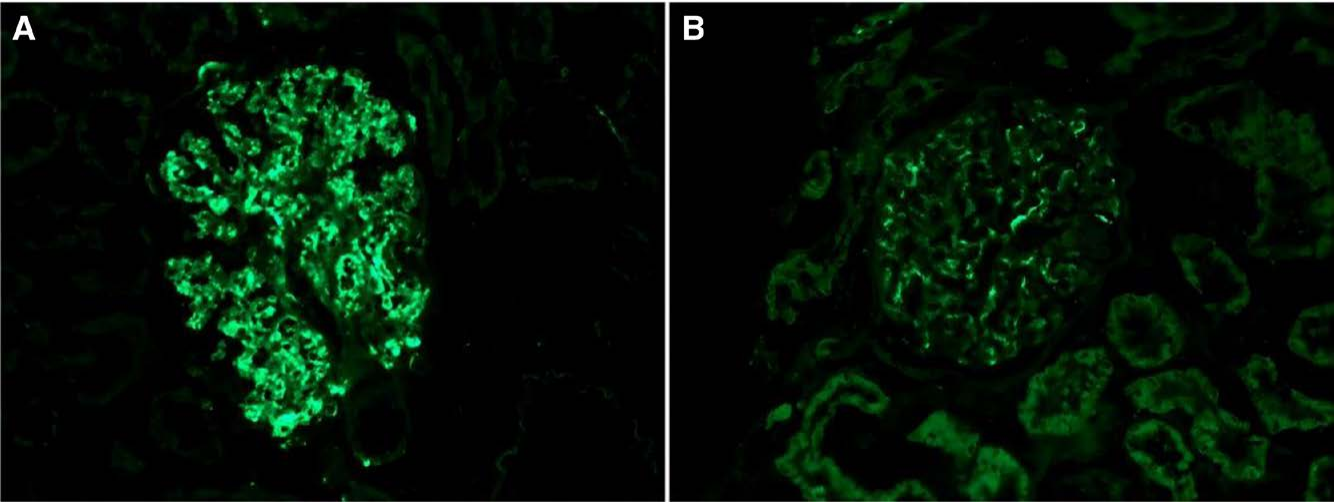
(A) Granular C3c deposition along the capillary wall and in the mesangium (indirect immunofluorescence staining on frozen tissue, 400×). (B) Granular IgA deposition along the capillary wall and in the mesangium (indirect immunofluorescence staining on frozen tissue, 400×).

**Figure 2.**
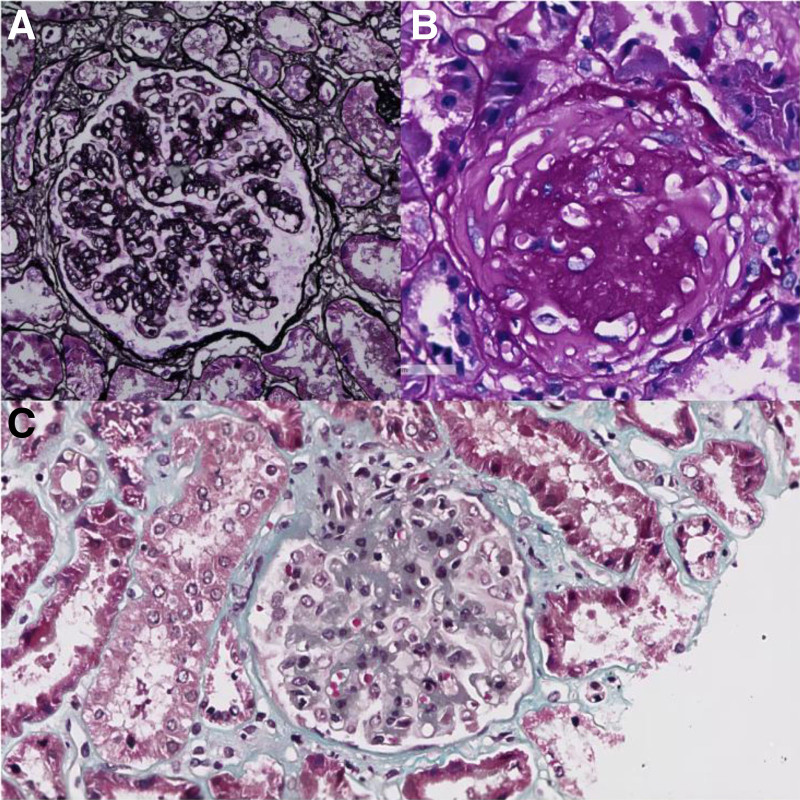
(A) Subcutaneous eosinophils were deposited in the mesangial area, the capillaries are lobulated, the capillary lumen is compressed, narrow and occluded, the lumen of some renal tubules was dilated, and the area of focal atrophy was about 5% to 10% (periodic acid-silver metheramine, 200×). (B) Glomeruli globally sclerosed, renal tubular epithelial cells were severely vacuolar and granular degeneration (periodic acid staining, 400×). (C) Glomerular mesangial cells and stroma are moderately and severely diffusely proliferated (Masson, 200×).

Two weeks later after hospitalization, we performed renal biopsy. The delay of renal biopsy was due to menstruation. Immunofluorescence examination revealed bright granular staining for C3 (++++), weak staining for IgA (++), kappa (+), lambda (+), but negative staining for IgG and C1q (Fig. 1). Endothelial cells showed vacuolar degeneration, focal endothelial cell proliferation and capillary loop compression. The wall layer of renal capsule was thickened and stratified, and the parietal cells proliferated with vacuolar degeneration. Segmental thickening of basement membrane and segmental insertion of mesangium. The epithelial cells of visceral layer were swollen, vacuolar degeneration, and foot processes were diffusely fused. Light microscopic examination exhibited that 6/19 glomeruli were globally sclerosed. Glomerular mesangial cells and stroma are moderately and severely diffusely proliferated, the capillaries are lobulated, the capillary lumen is compressed, narrow and occluded, a small amount of neutrophil infiltration can be seen in the lumen, the basement membrane is thickened, and the mesangium is visible. The matrix was inserted, the double track sign was formed, the subcutaneous eosinophils were deposited in the mesangial area, there was no obvious proliferation of parietal epithelial cells, and no crescent was formed. The renal tubular epithelial cells were severely vacuolar and granular degeneration. The lumen of some renal tubules was dilated, and the area of focal atrophy was about 5% to 10%. There was small focal inflammatory cell infiltration in renal interstitium, and there was no obvious lesion in the wall of small artery (Fig. 2).

**Figure 3.**
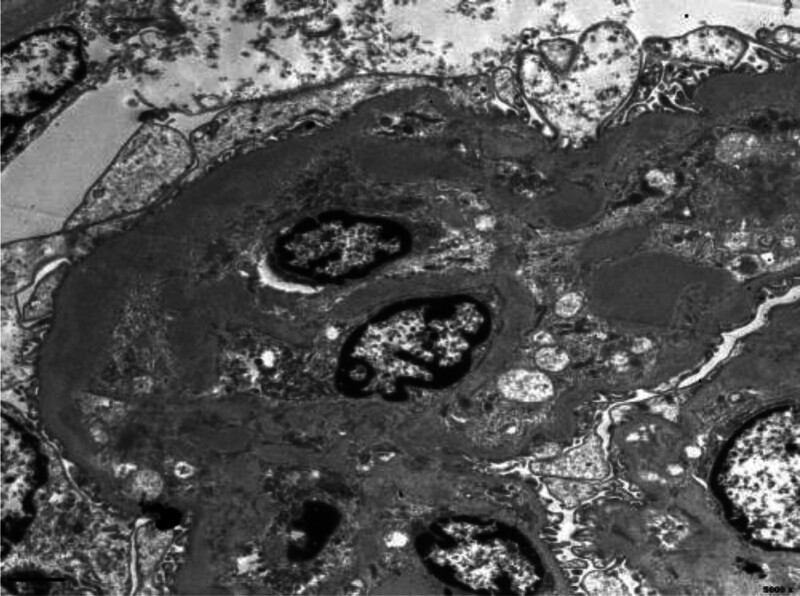
Electron dense deposits were found in the subendothelial and mesangial regions under EM (6000×).

One glomerulus was detected under electron microscope. Capillary loops were lobulated, endothelial cells were vacuolar degeneration, focal endothelial cell proliferation and capillary loop compression. The wall layer of renal capsule was thickened and stratified, and the parietal cells proliferated with vacuolar degeneration. Basement membrane shows segmental thickening, thickness of about 400 to 700 nm, segmental mesangial insertion. Visceral epithelial cells showed epithelial cells swelling, vacuolar degeneration. The foot processes were diffusely fused. Proliferation of mesangial cells and stroma observed in mesangial area. Electron dense deposits were found in the subendothelial and mesangial regions (Fig. 3).

An integrated screening of genes from the complement, coagulation and endothelial systems by using targeted genomic enrichment and massively parallel sequencing (Guangzhou Kingmed Center For Clinical Laboratory, China) revealed 3 heterozygous common variant of C3 (NM_000064.3: chr19:6697380; c.2771G > A; p.Gly924Asp), CD46 (NM_002389. 4: chr1: 207925586; c.29C > T; p.Pro10Leu), CD46 (NM_002389.4: chr1:207940537; c.853A > G; p.Lys285Glu).

The patient was worried about the side effects of drugs and strongly refused to use glucocorticoids and immunosuppressants, so she was given angiotensin II receptor blocker monotherapy for glomerulonephritis and quinolones for urinary tract infection. One month after discharge, the patients found that urinary tract infection was better than before. Urine routine showed that urinary protein (2+) and occult blood (3+) which were similar to those in hospital and complement C3 0.24 g/L. It seems that with the improvement of urinary tract infection symptoms, complement C3 increased, but hematuria and proteinuria did not change significantly.

## 3. Discussion

Our patient’s renal biopsy exhibited C3c-dominant deposition on immunofluorescence and though had weak staining for IgA. Complement alternative pathway was over-activated with normal plasma C4 levels and decreased plasma C3 levels. Thus, a definitive diagnosis of C3G was made. The electron microscopy further excluded the subtype of DDD, giving this patient a diagnosis of C3GN with an MPGN pattern.

The diagnosis of C3G depends on pathology, the evidence of abnormal activation of complement pathway, and the clinical characteristics of prolonged course and recurrent attack. The clinical manifestations of C3G are mainly characterized by proteinuria and/or hematuria of varying degrees, which are nonspecific. In most patients, serum complement C3 is decreased while complement C4 is normal, and the renal tissue immunofluorescence is mainly C3 deposition. These characteristics can help to exclude some diseases with similar clinical manifestations, such as IgA nephropathy, lupus nephritis, cryoglobulinemia, and renal damage. The overall prognosis of C3G is poor. Due to the lack of large-scale observation on the long-term prognosis of C3GN patients, it has been reported that C3GN progresses rapidly with poor prognosis. 36% to 50% of patients progress to end-stage renal disease within 10 years.^[1]^ The fluorescence intensity of C3 in pathology is inversely proportional to the prognosis of C3G.^[3]^ Generally speaking, the prognosis of C3GN is better than that of DDD. The current clinical observation mainly comes from non MPGN type C3GN, whose short-term prognosis is good, but the long-term prognosis may be poor; the differences reported may be related to the region, race and different diagnostic criteria.^[4]^ C3GN has multiple clinical and pathologic phenotypes. The varied therapeutic strategies and prognoses are attributed to the different pathogeneses. Thus, it is important to identify the precise causes after the diagnosis of C3GN in order to guide individualized treatment. We conducted gene screening tests for this patient. As a result, she was found to harbor multiple genetic changes, including common variants of C3 and CD46.

Variants in the C3 and CD46 genes were most frequently detected among the C3G patient. C3 plays a central role in the activation of classical and alternative complement activation pathways. It has been reported that C3 mutation can produce immune effect on inhibitory regulatory factors, and enhance disease activity in disguise.^[5,6]^ The protein encoded by CD46 is a type I membrane protein and is a regulatory part of the complement system. The encoded protein has cofactor activity for inactivation of complement components C3b and C4b by serum factor I, which protects the host cell from damage by complement. Mutant C3 showed a reduced binding to CD46 led to a decrease in proteolytic inactivation of C3b which may be a part of molecular mechanism of pathogenesis atypical hemolytic uremic syndrome.^[7,8]^ It has also been reported that CD46 enhances the internalization of *E coli* in urinary tract infection, making chronic urinary tract infection difficult to recover.^[9]^

The therapy for the C3G is variable due to no randomized clinical trials for treatments, though some recommendations were made to follow but none has been proven effective and beneficial, mainly including glucocorticoid combined immunosuppressive agents (such as mycophenolate mofetil, tacrolimus, etc).^[10,11]^ Biological agents (such as rituximab, ekuzumab),^[12]^ plasma infusion,^[13]^ and the development of new drugs (such as C3 invertase inhibitor cp40, recombinant human factor H, factor B inhibitor).^[14–16]^ It has been reported that factor B inhibitors shows a significant benefit for C3G in phase 2 clinical trials, phase 3 clinical trials are under way.

The clinical manifestations of this patient were persistent hematuria and moderate proteinuria which lasted for more than 8 years, besides there was a long history of chronic urinary tract infection. Before hospitalization, she had a history of upper respiratory tract infection. Her renal function was in normal range and C3 was significantly decreased. Combined with clinical symptoms and laboratory indicators, we cannot exclude infection-related glomerulonephritis (IRGN). After hospitalization, urine culture showed gram-negative bacteria infection. At present, most IRGN was caused by gram-positive bacteria, and a few were caused by gram-negative bacteria.^[17]^ One case reported a patient who is a 13-year-old female with Henoch–Schönlein purpura and similar symptoms: a history of recurrent urinary tract infection, diagnosed glomerulonephritis after *Campylobacter jejuni* infection.^[18]^ Unlike the 13-year-old female patient, our patient had no obvious gastrointestinal symptoms. Another case reported a 65-year-old patient with stable allograft function and *E coli* urosepsis. Renal biopsy finding revealed IgA-dominant postinfectious glomerulonephritis caused by *E coli* infection.^[19]^ Our patient’s urine culture showed *E coli* infection too, but renal biopsy showed C3GN not IRGN.

In the process of clinical diagnosis and treatment, C3GN should be distinguished from IRGN, including streptococcal infection associated glomerulonephritis and staphylococcal infection associated glomerulonephritis, because it is very similar to C3GN in clinical manifestations, pathological morphology and complement activation form.

Streptococcal infection associated glomerulonephritis is more common in children with acute nephritis syndrome and decreased complement C3. The typical renal pathological manifestations can be seen as proliferative glomerulonephritis in capillaries under light microscope. Immunofluorescence often has bright C3 deposition with or without IgG deposition. Under electron microscopy, more “Hump” like substances can be seen in subepithelial deposition. The clinical course of glomerulonephritis after streptococcal infection is often self-limiting. The level of complement C3 usually recovers naturally in 8 to 12 weeks, and the prognosis is good. A small number of patients cannot recover, or even progress to the end stage. For those who got streptococcal infection, C3 glomerulopathy should be noted with persistent renal injury and continuous decrease of complement. It has been reported that glomerulonephritis can be transformed into C3 nephropathy after *Streptococcus* infection.^[20]^

Staphylococcal infection associated glomerulonephritis is more common in middle-aged and elderly patients. Unlike post infection nephritis, *Staphylococcus* (common *Staphylococcus aureus*) infection associated glomerulonephritis and infection occur at the same time. Clinical manifestations include hematuria, varying degrees of proteinuria and renal dysfunction. Most of the patients were accompanied by hypocomplementation. The pathological manifestations of kidney were mainly C3 or co-deposition with IgA or IgG in the kidney. Most of the patients showed capillary hyperplasia or exudative lesions under light microscope. With the removal of infection foci in most patients, renal disease was alleviated, but the prognosis of kidney disease was worse than that of Staphylococcal infection associated glomerulonephritis. The key to differentiate from C3G is that complement C3 in C3G often decreases continuously, while the decrease of C3 level in patients with staphylococcal infection associated glomerulonephritis is often temporary. With the control of infection and the improvement of disease condition, C3 level can return to normal.^[17,21]^

It is certain renal biopsy still plays an important role in the diagnosis of kidney disease. In this case, there is no direct evidence that C3GN is caused by gram-negative bacteria associated infection, despite an interesting phenomenon that with the remission of urinary tract infection symptoms, complement C3 increased, but whether there is a direct relationship between chronic infection of gram-negative bacteria and C3GN with a CD46 variation is still uncertain. The patient refused to use glucocorticoid and immunosuppressant, and was treated with angiotensin II receptor blocker. With the patient is under follow-up.

## Acknowledgments

The authors wish to thank the patient who participated in this study.

## Author contributions

**Supervision:** Junfen Fan.

**Writing – original draft:** Chao Xu.
